# Altered Gut Microbiota Composition Is Associated With Back Pain in Overweight and Obese Individuals

**DOI:** 10.3389/fendo.2020.00605

**Published:** 2020-09-02

**Authors:** Marloes Dekker Nitert, Aya Mousa, Helen L. Barrett, Negar Naderpoor, Barbora de Courten

**Affiliations:** ^1^School of Chemistry and Molecular Biosciences, The University of Queensland, St Lucia, QLD, Australia; ^2^Monash Centre for Health Research and Implementation, School of Public Health and Preventive Medicine, Monash University, Clayton, VIC, Australia; ^3^Department of Endocrinology, Mater Hospital, Mater Misericordiae Ltd., South Brisbane, QLD, Australia; ^4^Mater Research Institute, The University of Queensland, South Brisbane, QLD, Australia; ^5^Department of Medicine, School of Clinical Sciences, Monash University, Clayton, VIC, Australia

**Keywords:** gut microbiota, back pain, *Adlercreutzia*, obesity, inflammation

## Abstract

**Background:** Back pain is the leading cause of disability worldwide and is associated with obesity and chronic low-grade inflammation. Alterations in intestinal microbiota may contribute to the pathogenesis of back pain through metabolites affecting immune and inflammatory responses.

**Aims and Methods:** We compared the gut microbiota composition in a cohort of 36 overweight or obese individuals with or without self-reported back pain in the preceding month. Participants were characterized for anthropometry; bone health; metabolic health; inflammation; dietary intake; and physical activity.

**Results:** Demographic, clinical, biochemical characteristics, diet and physical activity were similar between participants with (*n* = 14) or without (*n* = 22) back pain. Individuals with back pain had a higher abundance of the genera *Adlercreutzia* (*p* = 0.0008; FDR = 0.027)*, Roseburia* (*p* = 0.0098; FDR = 0.17), and *Uncl. Christensenellaceae* (*p* = 0.02; FDR = 0.27) than those without back pain. *Adlercreutzia* abundance remained higher in individuals with back pain in the past 2 weeks, 6 months, and 1 year. *Adlercreutzia* was positively correlated with BMI (rho = 0.35, *p* = 0.03), serum adipsin (rho = 0.33, *p* = 0.047), and serum leptin (rho = 0.38, *p* = 0.02).

**Conclusions:** Our findings suggest that back pain is associated with altered gut microbiota composition, possibly through increased inflammation. Further studies delineating the underlying mechanisms may identify strategies for lowering *Adlercreutzia* abundance to treat back pain.

## Introduction

Back pain is the single leading cause of disability worldwide and poses a considerable health and financial burden (1). Globally, back pain affects around 12% of the adult population, with a 1-month, 1-year, and lifetime prevalence of 23, 38, and 40%, respectively (2). These figures are expected to rise in line with an aging and more obese population (2). Obesity in particular is a known risk factor for back pain, but it is unclear whether this is due to mechanical stress on spinal structures or more systemic factors (3). Evidence suggests that chronic low-grade inflammation, which often co-exists with obesity, may contribute to back pain (4). Indeed, we (5) and others (6) have previously reported elevated cytokines and adipokines in overweight or obese individuals with back pain compared to those without back pain. An improved understanding of the mechanisms and potential triggers for back pain is therefore critical to facilitate primary prevention and inform future therapies as there are few effective treatments (7).

The gut microbiota (i.e., the composite of microorganisms present in the gastrointestinal tract) is fundamental for various aspects of host physiology including development, metabolism, and immunity, and can contribute to inflammation and pain (8). Germ-free mice are more sensitive to visceral pain, have altered pain perception regions in the brain and increased inflammation in the spinal cord (9), indicating the importance of the gut microbiota in pain perception. In addition, in chemotherapy-induced neuropathic pain, the gut microbiota plays a critical role through stimulation of the lipopolysaccharide—toll-like receptor pathway in rodent models (10). A recent meta-analysis has shown that gut microbiota composition is altered in obese individuals (11). These alterations, occurring through diet, disease, or other environmental influences, can cause dysbiosis—a state characterized by an overgrowth of potentially pathogenic organisms (pathobionts) (12). Imbalanced pathobiont/ symbiont composition reduces intestinal barrier integrity and function, promoting chronic inflammation and potentially inducing pain (13). Dysbiosis of the gut microbiota has also been implicated in the pathogenesis of various diseases, including inflammatory bowel syndrome, cancer as well as autoimmune diseases including rheumatoid arthritis and celiac disease (14–18). More recently, dysbiosis has been indirectly associated with the development of osteoarthritis (19, 20), visceral pain, and central nervous system functioning (21, 22) through altering the risk factors for these conditions.

Given this evidence, it is possible that back pain in obesity is driven by a persistent state of chronic low-grade inflammation partially driven by dysbiosis of the gut microbiome. On the other hand, restoring a healthy microbial community could potentially prevent or treat back pain through reducing systemic inflammation. While this is an intriguing hypothesis, there are currently no studies examining whether gut microbiota composition is altered in individuals who suffer from back pain and the potential mechanisms underlying these interactions.

To address this knowledge gap, we performed an exploratory study utilizing data from a well-characterized cohort of overweight or obese, otherwise healthy individuals to determine if gut microbiota composition differs in individuals with or without self-reported back pain.

## Methods

### Study Design and Population

This cross-sectional study utilizes baseline data from a cohort of 65 non-diabetic overweight or obese participants who took part in a randomized controlled trial examining the effects of vitamin D supplementation on cardiometabolic risk factors (23). Participants were advised of the primary purpose of the RCT (examining the effects of vitamin D on cardiometabolic risk), but also consented to the samples and data being stored and used for further sub-studies. Thirty-six individuals consented to providing stool samples for gut microbiota analyses and who had complete data for back pain were included in the present study. Participants were not selected into the cohort based on back pain status, but were included if they met the following inclusion criteria: age between 18 and 60 years; BMI ≥ 25 kg/m^2^; no weight changes ≥5 kg within the last year or intention for weight loss during the study; and no known or newly diagnosed co-morbidities based on medical history, physical exam, and routine blood tests. Participants were excluded if they: were smokers or had alcohol consumption exceeding four standard drinks/week for men and two standard drinks/week for women; had a diagnosis of diabetes (known or newly diagnosed), any other medical condition including injury or chronic or degenerative diseases, recent or current (acute or chronic) inflammation based on clinical history or blood tests; used any supplements or medications including pain medications, or anti-inflammatory, antibiotic, or psychotropic medications; or were pregnant, lactating or peri- or post-menopausal.

### Ethics

All participants provided written informed consent before enrolling in the study. The study was approved by the Monash University and Monash Health Human Research Ethics Committees (CF13/3874-2013001988).

### Anthropometric, Clinical, and Self-Reported Measures Including Back Pain

Body mass index (BMI) was calculated as weight (kg)/ height (m)^2^, and waist and hip circumferences were used to determine waist-hip ratio as an additional index for body fat distribution [WHR = waist (cm)/hip (cm)]. Bone mineral density (BMD) was measured by dual energy X-ray absorptiometry (DEXA) scans and is expressed as g/cm^2^. All participants underwent a medical history and physical examination, as well as 75 g oral glucose tolerance tests to exclude diabetes using World Health Organization criteria (24) and hyperinsulinemic-euglycemic clamps to assess insulin sensitivity as previously described (23). As part of the baseline questionnaires, participants were asked to a respond to a question regarding whether they had experienced back pain in the past 2 weeks, 1 month, 6 months, and/or 1 year and responses were recorded as “yes” or “no” for each timepoint. Data from the 1 month timepoint were used to determine back pain status and group participants for the analyses in this study. Participants were asked to provide stool samples during the study visits where possible and these were stored immediately in −80°C freezers. Otherwise, participants were instructed to store the sample in the fridge if not delivered within 4 h of collection. All samples were transported to the laboratory using a specialized service with temperature-controlled packaging and dry ice and were thawed prior to analysis. Participants were also asked to complete validated questionnaires on physical activity and diet. The International Physical Activity Questionnaire (IPAQ) was used to calculate multiples of the resting metabolic rate (METs) to characterize the level of exercise intensity, wherein a single MET represents the energy utilized by the body at rest. Data on dietary intakes of participants were collected using 3-days food diaries (analyzed using Foodworks 8.0 Professional; Xyris Software).

### Biochemical Measures

Fasting venous blood samples were collected after a 12-hr overnight fast and analyzed under blinded conditions by accredited and quality-assured laboratories (Monash Health and Monash University Pathology Departments, Australia). Plasma high sensitivity C-reactive protein (hsCRP) concentrations were measured using highly sensitive near-infrared particle immunoassays on a Synchron LX system analyzer according to manufacturer's instructions (Beckman Coulter Inc., Australia). Inter- and intra-assay coefficients of variation (CVs) were <3% and <5%, respectively. A bead-based multi-analyte assay was used to simultaneously measure four adipokines: leptin, adiponectin, adipsin, and resistin (LEGENDplex™ Human Metabolic Panel; Biolegend, CA, USA). Data were analyzed using the LEGENDplex™ Data Analysis Software (BioLegend, CA, USA) with standard curves generated from 0 to 200 ng/ml and samples adjusted for dilution factors. All adipokines had inter-assay CVs < 8% and intra-assay CVs < 9%. Serum 25-hydroxyvitamin D concentrations were measured using direct competitive chemiluminescent immunoassays (DiaSorin Inc., MN, USA) with inter- and intra-assay CVs of <10% and <4%, respectively. Serum calcium and phosphate were measured using automated colorimetric assays on SYNCHRON LX and SYNCHRON DXC800® systems and serum intact parathyroid hormone (PTH) was measured using Access/DXI PTH assays, which are paramagnetic particle chemiluminescent immunoassays (Beckman Coulter Inc., Australia). Inter- and intra-assay CVs were <5, <3, and <9% for serum calcium, phosphate, and PTH, respectively.

### Gut Microbiota Sequencing and Analysis

Thawed stool (250 mg) was lysed using the repeated bead beating method (RBB+C) with a mixture of 0.1 and 0.5 mm sterile zirconia beads followed by DNA isolation with the Maxwell 16 blood purification kit (Promega, Madison, WI, USA). A water sample was included as a negative control in parallel with the isolation of DNA from the stool sample and included in all steps of the sample treatment. DNA quantity and quality were assessed with spectrophotometry (Nanodrop ND1000). The V6-V8 region of the *16S rRNA* gene was amplified using the 926F forward primer (5′- TCG TCG GCA GCG TCA GAT GTG TAT AAG AGA CAG AAA CTY AAA KGA ATT GRC GG−3′) and the 1392R reverse primer (5′–GTC TCG TGG GCT CGG AGA TGT GTA TAA GAG ACA GAC GGG CGG TGW GTR C-−3′). A sequencing library was produced by barcoding amplicons using the Nextera XT V2 Index kit set A (Illumina, San Diego, CA, USA) and purified with the AMPure XP beads (Beckman Coulter, Indianapolis, IN, USA). The libraries were quantified, normalized and pooled before sequencing in the Illumina MiSeq instrument in the Australian Center for Ecogenomics (ACE) at the University of Queensland. All samples were run in the same sequencing run on the MiSeq. The QIIME (Quantitative Insights Into Microbial Ecology) software suite version 1.9.1 was used to join, demultiplex and quality filter the sequencing reads. Sequences were assigned to operational taxonomic units (OTUs) using the Greengenes database with the open reference OTU picking method and a 97% pairwise identity threshold. From the lysis step onward, we processed a DNA extraction control and a PCR control in a manner identical to all other samples. The control samples were pooled and sequenced on the MiSeq. Sequencing reads were subjected to quality filtering and OTUs detected in the negative control samples were deleted from the OTU tables. All OTUs with an overall abundance below 0.0001 were excluded from further analysis. Data were normalized with the cumulative sum scaling method followed by log transformation.

### Statistical Analyses

All data, including the participant characteristics, are presented as median (interquartile range) given that microbiota data are not normally distributed. Participant characteristics were compared between the groups with Mann-Whitney *U*-tests. Interindividual (alpha diversity) and between group (beta diversity) analyses were performed on the gut microbiota composition using the Shannon and Chao1 indices for alpha diversity and with unsupervised (PCoA) and supervised (RDA) clustering for beta diversity using the Calypso software tool (25). Differences in microbiota composition were assessed by Kruskal-Wallis rank testing. Adjustment for multiple testing was performed with Bonferroni correction or False Discovery Rate analysis. Analyses of the core microbiota (bacterial taxa present in >40% of all individuals) in each group were performed by Kruskal-Wallis rank testing. Two-tailed *p* < 0.05 after Bonferroni correction were considered statistically significant. Correlation analyses between clinical parameters and the three differentially expressed bacterial taxa were conducted using Spearman's correlations (uncorrected for multiple testing due to small numbers).

## Results

Participant characteristics are described in Table 1. Thirty-six participants were included in the study, of which 22 (61%) were male and approximately half (*n* = 19; 53%) were classified as obese (BMI ≥ 30 kg/m^2^) while the remaining 17 (47%) were overweight (BMI ≥25 kg/m^2^). There were no differences in demographic, clinical, or biochemical characteristics of participants who had experienced back pain in the past month compared with those without back pain (Table 1). Dietary composition, including total energy intake and carbohydrate, fat, and protein contents of the diet also did not differ between groups (all *P* > 0.05; data not shown).

**Table 1:** Participant characteristics.

**Characteristic**	**No back pain in the past month** **(*n* = 22)**	**Back pain in the past month** **(*n* = 14)**	***P***
Age (years)	34 (25–42)	30 (27–36)	0.6
Males, *n* (%)	14 (64)	8 (57)	0.7
Ethnicity (Caucasian/Asian/Other)	2/17/3	4/8/2	0.3
Physical activity (IPAQ-METs score)	3,104 (1,180–4,868)	1908 (615–3,510)	0.4
BMI (kg/m^2^)	29.9 (28.0–32.4)	30.9 (28.2–34.5)	0.3
WHR	1.1 (0.8–1.4)	1.1 (0.9–1.2)	0.5
Insulin sensitivity (*M*, mg/kg/min)	5.7 (4.0–7.7)	5.6 (3.9–8.6)	0.8
Vitamin D (25(OH)D, nmol/L)	32.5 (22.8–42.8)	26.0 (18.5–34.3)	0.2
Calcium (mmol/L)	2.3 (2.3–2.4)	2.3 (2.2–2.4)	0.5
Phosphate (mmol/L)	1.1 (1.0–1.3)	1.0 (1.0–1.2)	0.1
PTH (pmol/L)	4.6 (4.1–6.2)	5.7 (4.1–6.6)	0.4
BMD (g/cm^2^)	1.2 (1.2–1.3)	1.3 (1.2–1.4)	0.3
hsCRP (mg/L)	1.6 (1.0–3.1)	3.7 (1.0–6.3)	0.2
Calprotectin (μg/g)	10.5 (4.9–28.5)	11.0 (4.9–35.5)	0.8
Adiponectin (μg/ml)	3.5 (1.5–8.1)	1.1 (2.0–22.4)	0.1
Leptin (ng/ml)	2.3 (1.5–9.2)	8.8 (1.5–26.4)	0.15
Adipsin (ng/ml)	690.4 (448.8–889.3)	1240.0 (600.5–3073.0)	0.06
Resistin (ng/ml)	0.5 (0.3–1.0)	0.4 (0.3–1.2)	0.8

*Data are presented as median (IQR), all comparisons made with Mann-Whitney U-tests. BMD, bone mineral density; BMI, body mass index; hsCRP, high-sensitivity C-reactive protein; IPAQ-METs, international physical activity questionnaire- multiples of the resting metabolic rate; PTH, parathyroid hormone; WHR, waist-hip ratio*.

### Differences in Alpha and Beta Diversity by Back Pain Status

**Figure 1:**
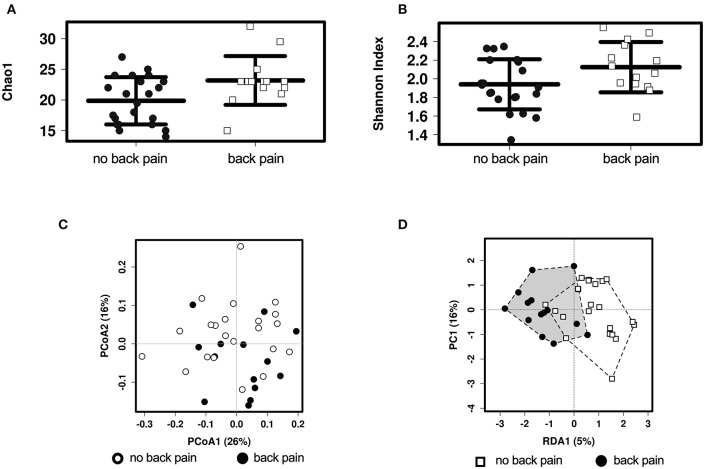
Alpha and Beta diversity analyses of the associations between gut microbiota and back pain. Alpha diversity in individuals with and without back pain in the past month as assessed by the Chao1 **(A)** or the Shannon **(B)** index. Beta diversity as assessed by unsupervised PCoA analysis **(C)** and by supervised RDA analysis **(D)** with the black and white shapes representing individuals with and without back pain, respectively.

A total of 154 OTUs were identified in the microbiome of this cohort, corresponding to 64 different genera of bacteria. The diversity of the gut microbiota composition was compared between groups. The alpha diversity (i.e., the diversity of the gut microbiota within an individual) was higher in people who experienced back pain in the past month when assessed with the Chao1 index (Figure 1A, *P* = 0.0018) and with borderline significance when assessed with the Shannon index (Figure 1B, *P* = 0.05). The difference between the Chao1 and Shannon indices is that Chao1 only takes into account abundance whereas the Shannon index also adjusts for evenness between bacterial genera. When examining differences between the groups of participants (beta diversity), there were no differences when comparing the groups in an unsupervised clustering analysis (Figure 1C, *P* > 0.05). However, in a supervised clustering analysis, the groups could be separated (Figure 1D, *P* = 0.03).

### Differences in Microbiota Abundance by Back Pain Status

**Figure 2:**
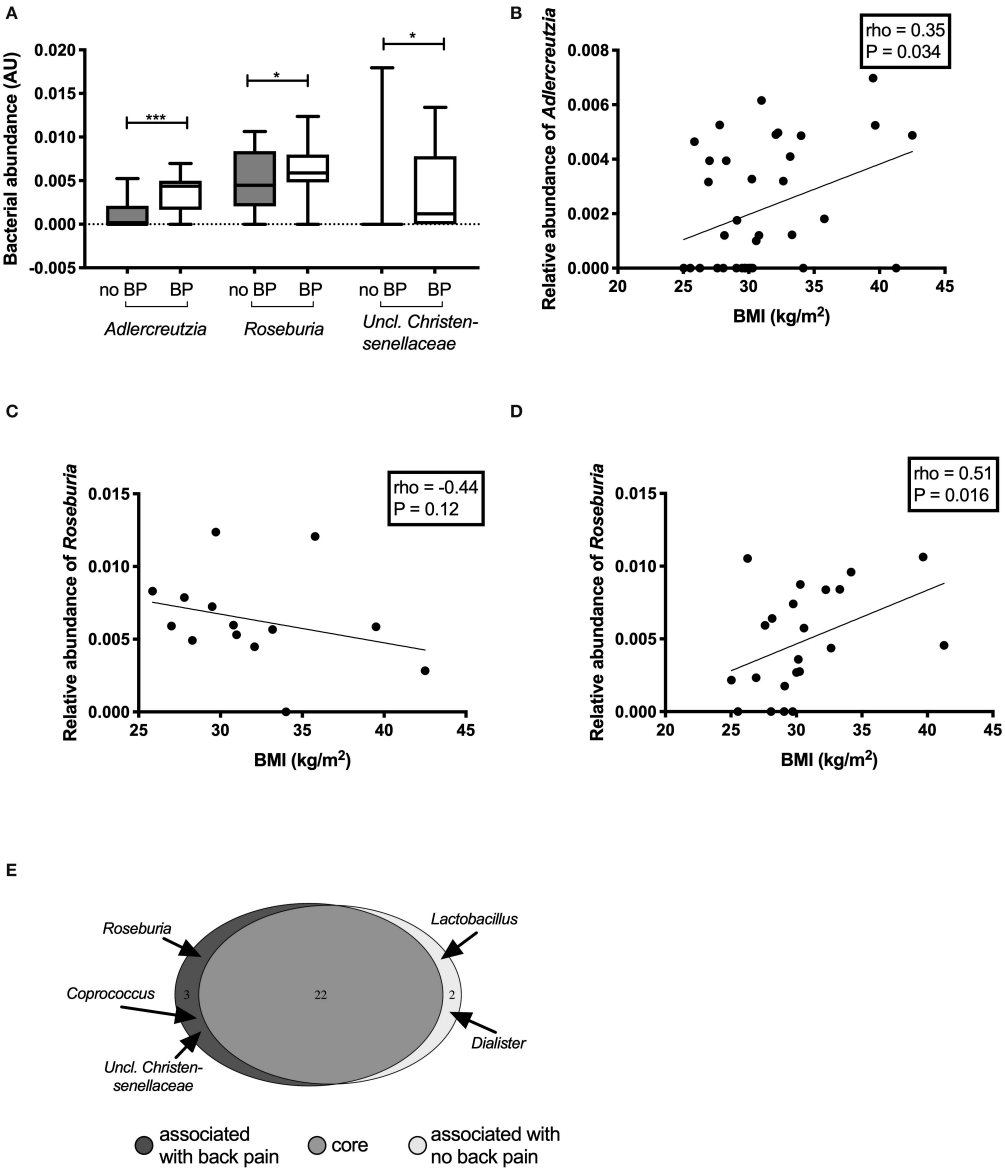
Differentially abundant genera between individuals with and without back pain in the past month. Genera that were found to be significantly altered in the gut microbiota of individuals with back pain (BP) **(A)**. Correlations between the abundance of the genus *Adlercreutzia* and BMI **(B)** in all participants. Correlations between the abundance of the genus *Roseburia* and BMI in participants with back pain **(C)** and without back pain **(D)**. Bacteria that made up the core microbiome of the individuals in this study **(E)**.

Participants who experienced back pain in the past month had significantly higher abundance of *Adlercreutzia* (*P* = 0.0008), *Roseburia* (*P* = 0.0098), and *Uncl. Christensenellaceae* (*P* = 0.02) (Figure 2A) compared with those without back pain. *Adlercreutzia* abundance remained significantly higher in individuals with back pain after correction for multiple testing (P_adj_ = 0.03, FDR = 0.03), whereas differences in *Roseburia* or *Uncl. Christensenellaceae* were attenuated (P_adj_ = 0.3, FDR = 0.17, and P_adj_ = 0.8, FDR = 0.27, respectively).

In analyses of back pain at other timepoints including back pain in the past 2 weeks (*n* = 12), 6 months (*n* = 17), or 1 year (*n* = 21), *Adlercreutzia* abundance was higher in participants with back pain compared with those without back pain (all *P* < 0.001; Supplementary Figure 1). Differences in *Roseburia* and *Uncl. Christensenellaceae* were only present when back pain was assessed over the past month but not at the other timepoints.

### Correlations Between Bacterial Abundance and Anthropometric, Clinical, and Biochemical Measures

The abundance of *Adlercreutzia* was positively correlated with BMI (rho = 0.35, *P* = 0.03; Figure 2B). *Adlercreutzia* abundance was also positively correlated with circulating adipsin (rho = 0.33, *P* = 0.04; Supplementary Figure 2A) and leptin concentrations (rho = 0.38, *P* = 0.02; Supplementary Figure 2B). There were no correlations between *Adlercreutzia* and BMD (rho = 0.10, *P* = 0.57), insulin sensitivity (rho = −0.09, *P* = 0.61), serum calcium (rho = −0.16, *P* = 0.34), phosphate (rho = 0.07, *P* = 0.68), or PTH levels (rho = 0.005, *P* = 0.98).

*Roseburia* abundance in the overall cohort was not correlated with any of the clinical or biochemical characteristics tested. However, when the correlation between *Roseburia* and BMI was examined separately in the group with or without back pain, the relationship was altered by the presence of back pain. In the group with back pain, *Roseburia* abundance tended to negatively correlate with BMI but this did not reach statistical significance (rho = −0.44, *P* = 0.1) (Figure 2C) whereas in the group without back pain, *Roseburia* abundance was positively correlated with BMI (rho = 0.51, *P* = 0.016) (Figure 2D). *Roseburia* abundance did not correlate with BMD (rho = −0.10, *P* = 0.58), insulin sensitivity (rho = −0.07, *P* = 0.69), serum calcium (rho = −0.20, *P* = 0.23), serum phosphate (rho = −0.04, *P* = 0.82), or PTH levels (rho = 0.19, *P* = 0.25). Given the small number of participants with *Uncl. Christensenellaceae* in their gut microbiota (*N* = 9, 25%), no correlation analyses were performed for this genus.

### Differences in Core Microbiota by Back Pain Status

We then performed an analysis of the core microbiota between groups. To be part of the core microbiota, the bacterium needs to be present in ≥40% of the participants in each group. The great majority of the genera (*n* = 22) were present in more than 50% of participants in each group (Figure 2E, Supplementary Table 1). Two genera were specific to the core microbiota of those participants without back pain: *Dialister* (55 vs. 36%; median (IQR) abundance in sequence reads: no back pain 0.54 (0.0–1.80)% *vs*. back pain 0.0 (0.0–1.01)% of reads) and *Lactobacillus* [41 vs. 36%; sequence abundance no back pain 0.45 (0.15–1.17%) vs. back pain 0.32 (0.22–0.68)% of reads]. However, three bacterial genera were predominantly present in the gut microbiota of participants with back pain: *Roseburia* [36% of participants without back pain vs. 86% of those with back pain; sequence abundance no back pain 0.45 (0.22–0.81)% vs. back pain 0.59 (0.50–0.77)% of reads]; *Coprococcus* [27% vs. 43%; 0.83 (0.58–1.67)% vs. 1.10 (0.71–1.96)% of reads] and *Uncl. Christensenellaceae* [9 vs. 43%; 0.0 (0.0–0.0) vs. 0.12 (0.0–0.70)% of reads]. *Adlercreutzia* was part of the core microbiome of both groups but was present in 86% of participants with back pain in the past month compared to only 41% of those without. *Adlercreutzia* abundance was also higher in those with back pain [median (IQR) abundance 0.44 (0.23–0.59)% of reads than in those without (0.0 (0.0–0.16)%]. The differences in the proportion of individuals carrying *Adlercreutzia* in their gut microbiota between those with and without back pain were also present at the other timepoints. Using the 6-months timepoint, 82% of participants who had experienced back pain in the past 6 months had *Adlercreutzia* in their core microbiome compared to only 37% of those without back pain. Similarly, 81% of participants with back pain in the past year had *Adlercreutzia* as part of their core microbiome compared to 27% of those without back pain.

## Discussion

To our knowledge, this is the first study to examine the relationship between gut microbiota and back pain in humans. We found that the overall composition of the gut microbiota differs between individuals with self-reported back pain in the past month compared with those without back pain. Specifically, we report the novel finding that the abundance of *Adlercreutzia* was higher in individuals with back pain, and *Adlercreutzia* abundance was positively correlated with BMI and inflammation as measured by serum leptin and adipsin concentrations. We also found a higher abundance of *Roseburia* and *Uncl. Christensenellacea* in those with back pain in the past month but these differences were not observed at other timepoints and were attenuated after adjustment for multiple testing. Our findings suggest that *Adlercreutzia* may serve as a potential biomarker or therapeutic target for back pain in overweight or obese individuals and further studies to corroborate these findings are warranted.

**Figure 3:**
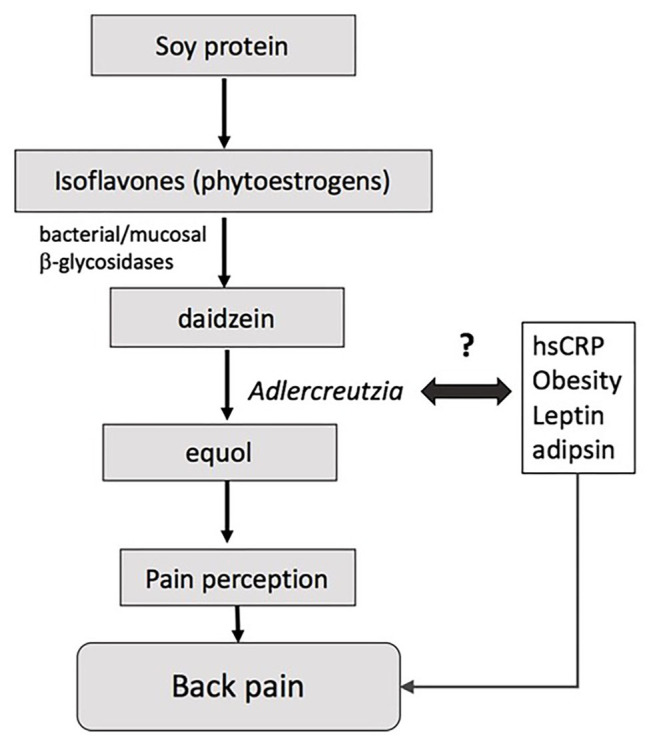
Model for how *Adlercreutzia* may influence back pain.

*Adlercreutzia* was present in most individuals experiencing back pain (86%) compared with less than half of those without back pain (41%), suggesting that the mere presence or absence of the bacteria, rather than its abundance *per se*, may drive the observed differences in back pain. *Adlercreutzia* is an obligately anaerobic coccobacillus belonging to the *Actinobacteria* phylum, and has non-spore forming, non-gas forming, and asaccharolytic properties (26). *Adlercreutzia* is known to play a role in the breakdown of isoflavones, which serve as phytoestrogens and are sourced primarily from dietary intake of soybeans (Figure 3). The first step in breaking down isoflavones is deglycosylation into daidzein and genistein by bacterial and mucosal β-glycosidases (27). *Adlercreutzia* is a bacterium that can further metabolize daidzein to equol (26). In humans, the capacity to produce equol is variable (28, 29) and equol has been shown to be critical in preventing bone loss as well as in improving muscle and joint pain in menopausal and postmenopausal women (30, 31). Moreover, phytoestrogens can directly modulate neuropathic pain via their antinociceptive properties, which are thought to be dependent upon the ability of the gut microbiota to degrade isoflavones (32). Previous studies have reported an altered capacity to degrade isoflavones or to metabolize diadzein to equol in obese individuals (29), and we speculate that this may be due to altered *Adlercreutzia* abundance. Taken together, these findings suggest that bone health and musculoskeletal pain may be influenced by an individual's intestinal flora for equol production and perhaps more specifically by the presence of *Adlercreutzia* (33, 34).

Higher *Adlercreutzia* abundance has also been correlated with lower circulating levels of non-essential amino acids including proline and alanine (35). These amino acids promote bone health and have been associated with higher BMD at the spine (36). Hence, the relationship between high *Adlercreutzia* abundance and back pain in our study may, in part, be due to low levels of these bone-protective amino acids. Our data does not fully support this hypothesis since we found no differences in BMD between participants with and without back pain, which may be due to the small sample size. Another putative mechanism by which intestinal bacterial species could contribute to back pain is via the development and/or maintenance of a chronic low-grade inflammatory state, which often co-exists with obesity. Indeed, we found that *Adlercreutzia* abundance was correlated with higher BMI, serum adipsin and leptin concentrations in the present cohort, but these correlations were not adjusted for multiple testing or potential confounders and should therefore be interpreted with caution. *Adlercreutzia* abundance has been linked to obesity before in rodents but not humans (37) and may be specifically associated with back pain. Nevertheless, a previous study by our group (5) found that higher adipsin and leptin concentrations were associated with back pain, and it is possible that these associations were driven by altered microbiota, specifically higher *Adlercreutzia*. Given that no previous studies have examined gut microbiota composition in relation to back pain, these explanations remain speculative and further studies are needed to elucidate the mechanisms by which these species may impact back pain.

We found that back pain in the past month was associated with *Roseburia* abundance; however, this was attenuated after adjustment for multiple testing. Abundance of the genus *Roseburia* has, to our knowledge, not been investigated with regard to pain. *Roseburia* is a known butyrate producer through fermenting dietary fiber (38). This may increase the caloric yield from food intake and therefore contribute to a higher BMI. In line with this, previous studies have shown that *Roseburia intestinalis* and related species are increased in people with higher BMI (38), which is consistent with our finding that *Roseburia* abundance was correlated with higher BMI in individuals without back pain. However, no correlations with BMI were found in the overall cohort or in those with back pain, suggesting that the relationship between *Roseburia* and BMI may be altered in individuals with back pain. It is not clear if and how butyrate produced by *Roseburia* contributes to back pain or if there are other metabolites such as hydrogen gas and lactate secreted by *Roseburia* (39) that could influence pain. Decreased levels of hsCRP have also been generally associated with increased *Roseburia* abundance (40), whereas in our cohort of participants with back pain, *Roseburia* was not associated with lower inflammation as measured by hsCRP. However, there are multiple different species and strains belonging to the *Roseburia* genus with different effects on metabolism and inflammation including some expressing high levels of pro-inflammatory flagellin proteins (41). It is therefore possible that individuals with back pain have increased abundance of these pro-inflammatory strains of *Roseburia*, although this was not captured by our sequencing approach.

Finally, we report a higher abundance of the genus *Uncl. Christensenellaceae* in individuals with back pain in the past month but not after adjustment for multiple testing. Contrary to this finding, a previous study of women with inflammatory bowel syndrome (42) found that a higher abundance of the family *Christensenellaceae* was associated with better quality of life and lower extra-intestinal pain (a composite of headache, back pain, joint pain and muscle pain), indicating that this family of bacteria may be associated with pain sensation. Our participants had no known inflammatory bowel syndrome, which has known significant effects on the composition of the gut microbiota (43). The family *Christensenellaceae* has also been reported to be negatively associated with obesity (11) and is increased in lean individuals (44, 45). In our study, the genus *Uncl. Christensenellaceae* was the only member of the family *Christensenellaceae* present in the gut microbiota of only 11 (31%) participants, hence we could not explore associations with the family *Christensenellaceae* or the relationship with BMI. Further studies are needed to establish whether *Uncl. Christensenellaceae* is associated with back pain independently of obesity.

The main limitations of this study include the relatively small size, the lack of information on the severity and frequency of the back pain and potential lack of statistical power to detect significant differences between groups (including in hsCRP which was twice as high in those with back pain, Table 1) or to maintain some associations after adjustment for multiple testing. In addition, back pain was not measured using a validated tool but was self-reported as a binary response by the participants and this could introduce bias in the data. Due to the cross-sectional design, we cannot establish causality or rule out reverse causality and it is possible that differences in gut microbiota composition are due to other factors besides back pain. For instance, we did not directly assess factors such as gastrointestinal symptoms, quality of life or psychological function, which may influence the results. While potential confounding factors could not be fully controlled, we attempted to minimize these by ensuring that the groups were similar in relevant demographic, clinical, biochemical, and lifestyle/dietary parameters which could influence microbiota composition and back pain. As per protocol (46), we studied overweight or obese non-diabetic adults (who were also vitamin D-deficient) and our results may not be generalizable to other populations including lean individuals or those with existing diseases. We used *16S rRNA* sequencing methods which may generate less reliable data at the species level compared with metagenomic sequencing approaches that would have also provided information on gene function. Notwithstanding these limitations, this is the first study assessing the composition of the gut microbiota in individuals suffering from back pain. Our sample comprised a well-characterized cohort of overweight or obese, otherwise healthy adults, where there was no confounding by disease status or use of medications (including pain medications), supplements, or substances, all of which can alter the gut microbiota composition and have seldom been excluded in previous studies.

## Conclusions

In summary, back pain in overweight healthy individuals is associated with altered gut microbiota composition, specifically higher abundance of the genera *Adlercreutzia, Roseburia*, and *Uncl. Christensenellaceae*. *Adlercreutzia* in particular showed a robust relationship with back pain, persisting across several timepoints and after adjustment for multiple testing. *Adlercreutzia* may represent a potential therapeutic target for back pain. However, this is an exploratory study and further large-scale investigations are needed to confirm our findings and to clarify the potential mechanisms by which these species could impact on back pain. An improved understanding of the role of microbiota in pain modulation can inform the development of novel therapies (e.g., diet, supplements, fecal transplants) to potentially alleviate the burden of back pain and its associated disabilities.

## Data Availability Statement

The datasets generated for this study will not be made publicly available; our ethics committee does not allow this.

## Ethics Statement

The studies involving human participants were reviewed and approved by Monash univesrsity ethics committee. The patients/participants provided their written informed consent to participate in this study.

## Author Contributions

MD performed data analysis and interpretation, contributed to the microbiome profiling and analysis, and co-wrote the first draft of the manuscript. AM performed data collection, co-wrote the first draft of the manuscript, contributed to data interpretation, reviewing, and editing the manuscript. NN performed data collection, contributed to reviewing, and editing the manuscript. HB contributed to reviewing and editing the manuscript. BC conceptualized the study, obtained funding, oversaw data collection and analysis, supervised data interpretation, writing and editing of the manuscript, chief investigator of the study, the guarantor of this work, and takes responsibility for data integrity and accuracy. All authors meet the ICMJE criteria for authorship and have provided substantial intellectual input and approved the final version for publication.

## Conflict of Interest

The authors declare that the research was conducted in the absence of any commercial or financial relationships that could be construed as a potential conflict of interest.
