# Supplementary material for: Altered Gut Microbiota Composition Is Associated With Back Pain in Overweight and Obese Individuals
Source: Front Endocrinol (Lausanne). 2020 Sep 2;11:605. doi: 10.3389/fendo.2020.00605 (PMC7492308; doi:10.3389/fendo.2020.00605)
Supplement: Supplementary file 1 [file Data_Sheet_1.PDF]

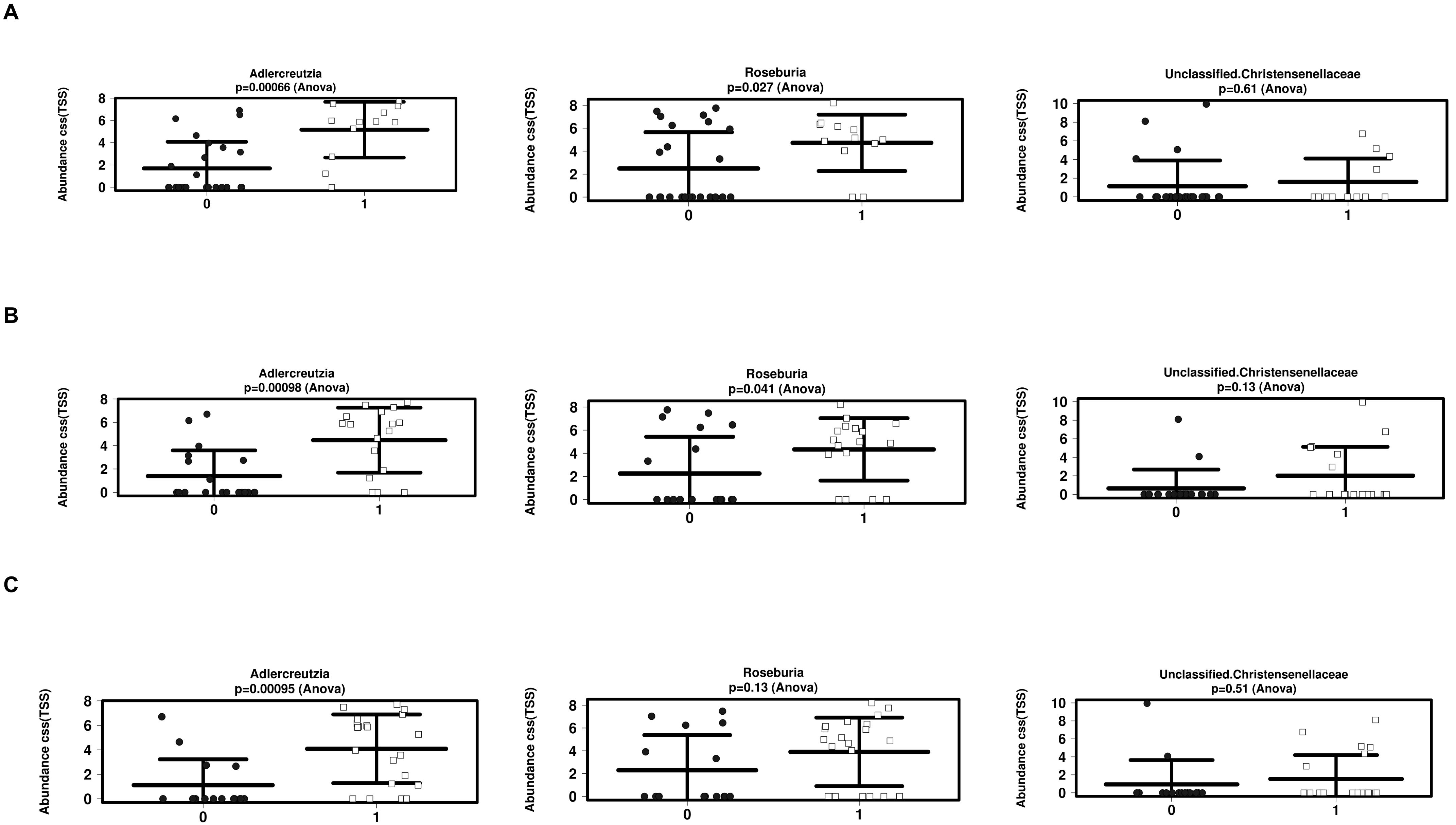

Supplementary Figure 1 Abundance of *Adlercreutzia* (left panels)*; Roseburia* (middle panels) and *Uncl. Christensenellaceae* (right panels) in people without (0, black circles) and with (1, white squares) backpain within the last two weeks (A), six months (B) and twelve months (C).
