# Supplementary figures and images for: Altered Gut Microbiota Composition Is Associated With Back Pain in Overweight and Obese Individuals
Source: Front Endocrinol (Lausanne). 2020 Sep 2;11:605. doi: 10.3389/fendo.2020.00605 (PMC7492308; doi:10.3389/fendo.2020.00605)

**A**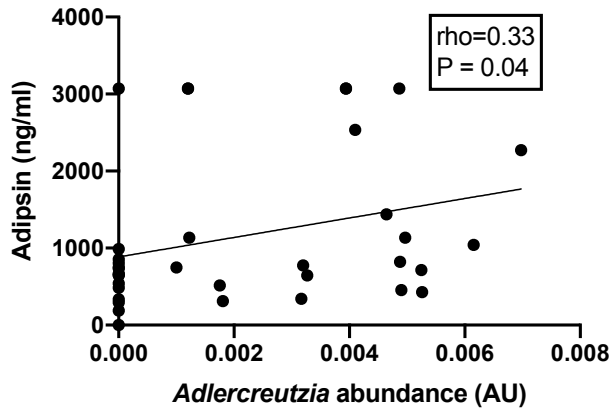**B**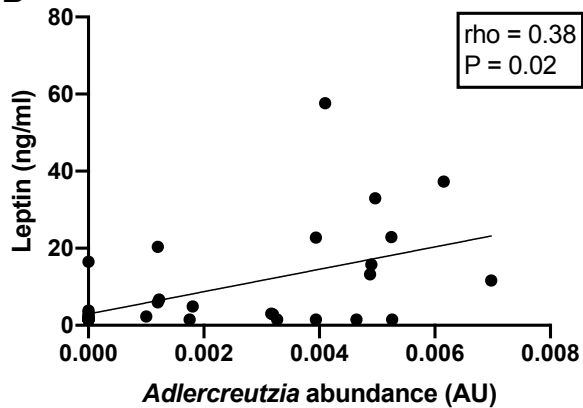

Supplement: Supplementary file 2 [file Data_Sheet_2.PDF]
